# Narrow-Margin Hepatectomy Resulted in Higher Recurrence and Lower Overall Survival for R0 Resection Hepatocellular Carcinoma

**DOI:** 10.3389/fonc.2020.610636

**Published:** 2021-01-21

**Authors:** Lihong Liu, Yongjie Shui, Qianqian Yu, Yinglu Guo, Lili Zhang, Xiaofeng Zhou, Risheng Yu, Jianying Lou, Shumei Wei, Qichun Wei

**Affiliations:** ^1^ Department of Radiation Oncology, Ministry of Education Key Laboratory of Cancer Prevention and Intervention, the Second Affiliated Hospital, Zhejiang University School of Medicine, Hangzhou, China; ^2^ Department of Radiology, the Second Affiliated Hospital, Zhejiang University School of Medicine, Hangzhou, China; ^3^ Department of Hepatobiliary Pancreatic Surgery, the Second Affiliated Hospital, Zhejiang University School of Medicine, Hangzhou, China; ^4^ Department of Pathology, the Second Affiliated Hospital, Zhejiang University School of Medicine, Hangzhou, China

**Keywords:** hepatocellular carcinoma, surgical margin, patterns of recurrence, prognosis, postoperative radiotherapy

## Abstract

**Purpose:**

To evaluate the impact of resection margin on recurrence pattern and survival for hepatocellular carcinoma (HCC) with narrow margin resection, with the aim to guide postoperative treatment.

**Materials and Methods:**

Two hundred forty HCC patients after curative hepatectomy between 2014 and 2016 were reviewed retrospectively. The cases were divided into narrow-margin (width of resection margin <1cm, n=106) and wide-margin (width of resection margin ≥1cm, n=134) groups based on the width of resection margin. Recurrence pattern, recurrence-free survival (RFS), and overall survival (OS) were compared between the above two groups. An additional cohort of nine cases with positive margin plus post-operative stereotactic body radiotherapy (SBRT) was also analyzed for the recurrence pattern.

**Results:**

Postoperative recurrence was found in 128 (53.3%) patients. The recurrence rate was significantly higher in narrow-margin group than that in wide-margin group (P=0.001), especially for the pattern of marginal recurrence (20.8 *vs*. 4.5%, P=0.003). The 1-, 2-, 3-year RFS rates for the narrow-margin and wide-margin groups were 55.8, 43.9, 36.9, and 78.7, 67.9, 60.2%, respectively, with significant difference between the two groups (P<0.001). Patients with narrow margin showed a tendency of decreased OS than those with wide margin (P<0.001). As comparison, the nine cases with positive margin treated with postoperative SBRT showed low recurrence rate and no marginal recurrence was found.

**Conclusion:**

Patients with narrow resection margin were associated with higher recurrence rate and worse survival than those with wide resection margin. These patients may benefit from adjuvant local treatment, such as radiotherapy.

## Introduction

Hepatocellular carcinoma (HCC) is the seventh prevalent malignancy worldwide (1). In China, HCC is the fourth common cancer and the third leading cause of cancer-related mortality (2). Although surgical excision is considered the standard treatment for resectable HCC (3), a high rate of postoperative recurrence was observed after partial hepatectomy, with a marginal recurrence rate up to 30% (4–6). Multiple factors are correlated to high postoperative recurrence, including tumor size, number, microvascular invasion (MVI), tumor capsule invasion, and resection margin status (5, 7–10).

Among these factors, resection margin has been widely evaluated for its effect on the long-term outcomes after resection, however, the conclusion remains controversial. A few studies have shown that an adequate resection margin is indispensable for long-term survival (11–14), but others found that a narrow resection margin does not detract from long-term outcomes (8, 9, 15). It is generally accepted that both surgical curability and postoperative hepatic function preservation are crucial for the successful treatment of patients with HCC. For instance, irregular hepatectomy or meso-hepatectomy were often recommended for centrally located HCCs, especially for those adjacent to the first or second porta hepatis systems (16, 17), but the resection margin is generally less than 1cm in order to meet the criteria both for cure and preservation of the adjacent major vessels simultaneously (18, 19). Therefore, a better understanding of the impact of width of resection margin on recurrence and survival helps to tailor adjuvant therapy against recurrence to improve long-term oncological outcomes.

In this study, based on a retrospectively collected database, we conducted a detailed analysis to reveal the effect of margin width on recurrence pattern and survival outcomes after hepatectomy, in HCC with narrow resection margin. If the rate of marginal recurrence is high, a postoperative local treatment such as radiotherapy might be useful to improve local control.

## Materials and Methods

### Patients

A total of 240 HCC patients in the Second Affiliated Hospital, Zhejiang University School of Medicine (SAHZU) who underwent hepatectomy between April 2014 to December 2016 were enrolled in this study based on the following inclusion criteria: 1) HCC confirmed by postoperative histology. 2) without neoadjuvant treatment before the first hepatectomy. 3) a complete removal of tumor confirmed by postoperative pathology. 4) Child-Pugh class A5, A6, or B7. 5) Eastern Cooperative Oncology Group performance status 0 or 1. 6) with postoperative imaging follow-up of more than 2 months. Those with distant metastasis and second primary tumor were excluded. The clinical information and follow-up data were collected from the electrical records. The tumor differentiation was graded according to the Edmondson and Steiner grading system (20). The MVI status was graded by the guidelines for the pathological diagnosis of primary liver cancer: 2015 update (21). Tumor staging was assessed according to the 8^th^ edition of guidelines of the American Joint Committee on Cancer (AJCC) (22). Tumor capsule invasion were judged by preoperative imaging. Tumor with smooth peripheral rim was defined as presence of capsule, while tumor with irregular or indistinct borders were defined as absence of capsule (23). This study was approved by Institutional Review Board of SAHZU (2020774).

### Surgical Procedure

Surgical margin was defined as the shortest distance from the edge of the tumor to the surface of liver transection (24). The cases were divided into narrow-margin (width of resection margin <1cm, n=106) and wide-margin (width of resection margin ≥1cm, n=134) groups. Resection of the liver equal to or larger than two Couinaud’s segments was considered major liver resection, and a resection smaller than this was considered minor resection. R0 resection was defined as no cancer cell was found on the surgical margin under microscope. Preoperative and postoperative imaging (contrast-enhanced MRI or CT scans) were used to assess the size and location of tumor, and width of resection margin.

### Recurrence

After curative R0 resection, patients were followed up in our hospital every month for 3 times, then every 3 months during the first 2 years and every 6 months during the next 3 years. Biopsy for the recurrent lesion was encouraged. Imaging evidence of tumor recurrence (suspicious new findings and progression of disease documented by serial imaging) was also accepted in patients who did not undergo biopsy. The recurrence pattern and the date of initial disease relapse was recorded when the first suspicious radiologic finding was initially identified. In terms of location of recurrence, two major categories were divided: intrahepatic recurrence and extrahepatic recurrence. Intrahepatic recurrence was subdivided into three patterns: 1) marginal recurrence, 2) intrahepatic single-nodule recurrence, 3) intrahepatic multiple-nodule recurrence. Marginal recurrence was defined as intrahepatic recurrence located less than 1 cm from the resection margin, regardless of any simultaneous recurrence in the distant liver remnant or extrahepatic sites.

### Positive Margin Cases with Postoperative Stereotactic Body Radiotherapy

Postoperative radiotherapy such as stereotactic body radiotherapy (SBRT) is not recommended for HCC with wide resection margin and its role in patients with narrow margin resection remains controversial. In our institution, postoperative SBRT is not introduced for HCC with R0 resection, therefore, there are not relevant data to demonstrate the significance of adjuvant SBRT in HCC patients with narrow resection margin. However, for patients with positive margin, adjuvant SBRT was used as an optional treatment for residual lesions following hepatectomy, if the patients possess appropriate performance status (ECOG 0-2) and enough liver function reserve. We compared the difference in recurrence pattern between positive margin HCC who received SBRT and those with R0 resection alone, with the aim to evaluate the role of SBRT in local control.

The tumor bed was marked by surgical clips for patients with positive margin during operation. The gross target volume (GTV) was defined as tumor residual and the tumor bed. The full extent of fluid cavity was not included intentionally. The internal target volume (ITV) was defined as the volumetric sum of GTVs in the multiple phases. The planning target volume (PTV) included ITV with 0.5 cm margin and was adjusted manually to minimize overlapping the gastrointestinal tract when needed. The other radiation treatment details, including dose-volume constrains to organs at risk (OAR), respiratory motion management, image guidance, and evaluation of toxicities after SBRT were referred to our previous article by Shui et al. (25).

### Statistical Analysis

The data are presented as the mean ± standard deviation for continuous variables. Comparisons between groups were performed using the chi-square test (or the Fisher’s exact test) for nominal variables, and the unpaired t test was used for continuous variables. Overall survival (OS) and recurrence-free survival (RFS) were evaluated by the Kaplan-Meier method and compared by the log-rank test, respectively. OS was calculated from the date of first radical hepatectomy to death for any cause. RFS was measured from the date of first hepatectomy to first recurrence. The Cox regression model was employed in univariate analyses. Surgical resection, tumor number, size, and variables with p value <0.01 in the univariable analyses were retained for the multivariable Cox analysis. All statistical analyses were performed using IBM SPSS Statistics for Windows (Version 23.0; IBM Corp., Armonk, NY) and GraphPad Prism 8.0 software (GraphPad Software Inc., San Diego, CA, USA). P<0.05 was considered statistically significant and indicated by bold values.

## Results

### Patients Characteristics

The follow-up ended on March 10, 2020. The median follow-up time was 55.2 months [95% confidence interval (CI) 52.2–58.2 months]. Totally 240 patients were included in the final analysis. Of which 106 were divided into the narrow-margin group, and 134 into the wide-margin group based on the width of resection. The patients included 208 (86.7%) male and 32 (13.3%) female cases with an average age of 57.3 (range 22–82) years old at first operation. None of the patients received radiotherapy pre- or post-operatively. A comparison of the baseline demographic and clinicopathological characteristics showed that more patients presented with higher ALT level (P=0.030), larger tumor size (P=0.003), absence of tumor capsule (P=0.027), longer operative time (P<0.001), larger operative blood loss (P=0.019), major liver resection (P<0.001), and more advanced pTNM stage (P=0.008) in the narrow-margin group than that in the wide-margin group (**Table 1**). In this analysis, TACE was administered to 57 patients (57/240, 23.8%), with 29 (29/134, 21.6%) cases from the wide-margin group, the other 28 (28/106, 26.4%) from narrow-margin group. The patient distribution of receiving TACE were largely comparable between the two groups. We further compared the baseline and outcome characteristics of all 240 R0 resection patients with or without postoperative transcatheter arterial chemoembolization (TACE) (**Supplementary Table 1**). The clinicopathological characteristics of patients receiving TACE were comparable to those without receiving TACE.

**Table 1:** Demographic and clinicopathological characteristics of wide-margin, narrow-margin, and positive-margin plus stereotactic body radiotherapy (SBRT) groups.

Variable	Wide-margin group	Narrow-margin group	*P*-Value	Positive-margin plus SBRT
	(n = 134)	(n = 106)	(Wide *vs.* narrow)	(n = 9)
Age ≤60 years old >60 years old	87 (64.9%) 47 (35.1%)	61 (57.5%) 45 (42.5%)	0.243	6 (66.7%) 3 (33.3%)
Gender Male Female	113 (84.3%) 21 (15.7%)	95 (89.6%) 11 (10.4%)	0.231	8 (88.9%) 1 (11.1%)
HBs Ag Positive Negative	108 (80.6%) 26 (19.4%)	75 (70.8%) 31 (29.2%)	0.075	7 (77.8%) 2 (22.2%)
Cirrhosis Yes No	100 (74.6%) 34 (25.4%)	74 (69.8%) 32 (30.2%)	0.407	9 (100.0%) 0 (0.0%)
Alcohol consumption Yes No	75 (56.0%) 59 (44.0%)	57 (53.8%) 49 (46.2%)	0.734	1 (11.1%) 8 (88.9%)
AFP ≤20 ng/ml >20 ng/ml	64 (47.8%) 70 (52.2%)	39 (36.8%) 67 (63.2%)	0.088	5 (55.6%) 4 (44.4%)
ALT ≤40 U/L >40 U/L	94 (70.1%) 40 (29.9%)	60 (56.6%) 46 (43.4%)	**0.030**	6 (66.7%) 3 (33.3%)
TBIL ≤17.1umol/L >17.1umol/L	87 (64.9%) 47 (35.1%)	72 (67.9%) 34 (32.1%)	0.626	5 (55.6%) 4 (44.4%)
ALB ≤35 g/L >35 g/L	11 (8.2%) 123 (91.8%)	11 (10.4%) 95 (89.6%)	0.563	1 (11.1%) 8 (88.9%)
PT% <75 75-100 >100			0.189	
	9 (6.7%)	6 (5.7%)		1 (11.1%)
	96 (71.6%)	66 (62.3%)		4 (44.4%)
	29 (21.6%)	34 (32.1%)		4 (44.4%)
Child-Pugh class A5 A6 B7			0.377	
	123 (91.8%)	93 (87.7%)		8 (88.9%)
	10 (7.5%)	10 (9.4%)		0 (0.0%)
	1 (0.7%)	3 (2.8%)		1 (11.1%)
Tumor size ≤5 cm >5 cm	89 (66.4%) 45 (33.6%)	50 (47.2%) 56 (52.8%)	**0.003**	5 (55.6%) 4 (44.4%)
No. of tumor Single Multiple	119 (88.8%) 15 (11.2%)	86 (81.1%) 20 (18.9%)	0.094	8 (88.9%) 1 (11.1%)
Edmondson grades I-II III-IV	104 (77.6%) 30 (22.4%)	78 (73.6%) 28 (26.4%)	0.469	5 (55.6%) 4 (44.4%)
Tumor capsule Present Absent	115 (85.8%) 19 (14.2%)	79 (74.5%) 27 (25.5%)	**0.027**	3 (33.3%) 6 (66.7%)
MVI classification			0.385	
M0	77 (57.5%)	49 (46.2%)		2 (22.2%)
M1	37 (27.6%)	36 (34.0%)		4 (44.4%)
M2	17 (12.7%)	18 (17.0%)		3 (33.3%)
Unclear	3 (2.2%)	3 (2.8%)		0 (0.0%)
Extent of resection Minor Major	88 (65.7%) 46 (34.3%)	47 (44.3%) 59 (55.7%)	**<0.001**	3 (33.3%) 6 (66.7%)
Operative time	172.4 ± 72.0	224.9 ± 99.0	**<0.001**	276.5 ± 83.6
Operative blood loss	276.2 ± 514.7	454.5 ± 608.7	**0.019**	210.0 ± 87.6
pTNM stage I II^*^ III IVA	64 (47.8%) 52 (38.8%) 3 (2.2%) 15 (11.2%)	30 (28.3%) 48 (45.3%) 7 (6.6%) 21 (19.8%)	**0.008**	1 (11.1%) 7 (77.8%) 0 (0.0%) 1 (11.1%)
Postoperative TACE Yes No	29 (21.6%) 105 (78.4%)	28 (26.4%) 78 (73.6%)	0.446	5 (55.6%) 4 (44.4%)

Values in parentheses are percentages and P<0.05 was indicated by bold values.

HBs Ag, hepatitis B surface antigen; AFP, alpha-fetoprotein; ALT, alanine aminotransferase; TBIL, total bilirubin; ALB, albumin; PT, prothrombin time; MVI, microvascular invasion; pTNM stage, pathologic TNM stage; TACE, transcatheter arterial chemoembolization.

^*^5 cases that T stage (T1b or T2) was undefined because of unclear MVI status were belonged in category of T2 stage.

### Patterns of Recurrence

**Figure 1:**
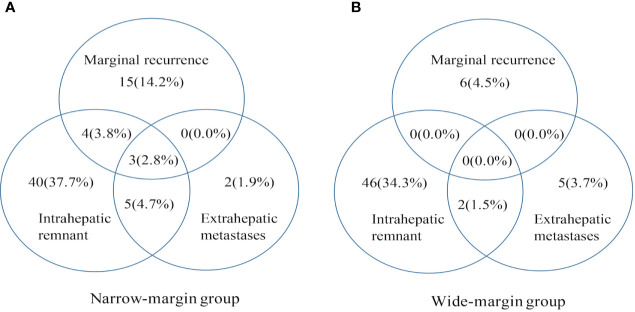
Patterns of initial recurrence for hepatocellular carcinoma (HCC) patients in narrow-margin and wide-margin groups. Patterns of initial recurrence stratified by marginal recurrence, intrahepatic remnant recurrence, and extrahepatic metastases. Values in parentheses are percentages. **(A)** Patterns of initial recurrence in the narrow-margin group. **(B)** Patterns of initial recurrence in the wide-margin group.

The median time to recurrence is 9.7 months. During the follow-up periods, 128 patients (128/240, 53.3%) had documented tumor recurrence, with 69 patients (69/106, 65.1%) were originally resected with a narrow margin, 59 (59/134, 44.0%) with a wide margin resection (P=0.001). The marginal recurrence rate was 20.8% (22/106) among patients in the narrow-margin group, and the corresponding rate was 4.5% (6/134) in the wide-margin group, with a significant difference between these two groups (P=0.003). The intrahepatic single-nodule recurrence rate was 12.3% (13/106) in the narrow-margin group, and the corresponding rate was 19.4% (26/134) in the wide-margin group, with a significant difference between the two groups (P=0.002). Intrahepatic multiple-nodule recurrences were found in 50 patients. Among them, 34 (34/106, 32.1%) were from the narrow-margin group, and 16 (11.9%) from the wide-margin group, with a significant difference between them (P=0.010). The recurrence patterns were summarized in **Table 2** and **Figure 1**, showing that the incidence of extrahepatic recurrence was low, while marginal recurrence and intrahepatic remnant recurrence were the main recurrence patterns.

**Table 2:** Patterns of recurrence of the wide-margin, narrow-margin, and positive-margin plus stereotactic body radiotherapy (SBRT) groups.

Variable	Wide-margin group	Narrow-margin group	*P*-Value	Positive-margin plus SBRT
	(N = 134)	(N = 106)	(Wide *vs.* Narrow)	(N = 9)
Total recurrence	59 (44.0%)	69 (65.1%)	**0.001**	2 (22.2%)
Type of recurrence				
Intrahepatic recurrence	54 (40.3%)	67 (63.2%)	0.321	2 (22.2%)
Extrahepatic recurrence	7 (5.2%)	10 (9.4%)	0.662	0 (0.0%)
Sites of intrahepatic recurrence				
Marginal recurrence	6 (4.5%)	22 (20.8%)	**0.003**	0 (0.0%)
Intrahepatic single-nodule	26 (19.4%)	13 (12.3%)	**0.002**	1 (11.1%)
Intrahepatic multiple-nodule	16 (11.9%)	34 (32.1%)^*^	**0.010**	1 (11.1%)
Unclear	6 (4.5%)	5 (4.7%)	0.556	0 (0.0%)
Time to recurrence				
Total recurrence				
~ 3 months (include 3)	11 (8.2%)	16 (15.1%)	0.530	0 (0.0%)
3~6 months	15 (11.2%)	30 (28.3%)	**0.033**	0 (0.0%)
6~9 months	20 (14.9%)	41 (38.7%)	**0.004**	2 (22.2%)
9~12 months	28 (20.9%)	46 (43.4%)	**0.028**	0 (0.0%)
Marginal recurrence				
~ 3 months (include 3)	7 (6.6%)	0 (0.0%)	**0.022**	0 (0.0%)
3~6 months	11 (10.4%)	1 (0.7%)	0.074	0 (0.0%)
6~9 months	13 (12.3%)	3 (2.2%)	0.164	0 (0.0%)
9~12 months	15 (14.2%)	3 (2.2%)	**0.033**	0 (0.0%)
Intrahepatic multiple-nodule				
~ 3 months (include 3)	10 (9.4%)	4 (3.0%)	0.252	0 (0.0%)
3~6 months	20 (18.9%)	6 (4.5%)	0.088	0 (0.0%)
6~9 months	25 (23.6%)	7 (5.2%)	0.057	1 (11.1%)
9~12 months	29 (27.4%)	9 (6.7%)	**0.010**	1 (11.1%)

^*^8 of them with concurrent marginal recurrence. P < 0.05 was indicated by bold values.

Compared with patients with wide margin resection, those with narrow margin resection had a higher rate of recurrence within 12 months after surgery (narrow and wide: 43.4 *vs*. 20.9%, P=0.028). More marginal recurrence occurred in narrow-margin group than wide-margin group at different time points of the first year after surgery (at 3 months: 6.6 *vs*. 0.0%; at 6 months: 10.4 *vs*. 0.7%; at 9 months: 12.3 *vs*. 2.2%; at 12 months: 14.2 *vs*. 2.2%). Similar results were also found for intrahepatic multiple-nodule recurrence (at 3 months: 9.4 *vs*. 3.0%; at 6 months: 18.9 *vs*. 4.5%; at 9 months: 23.6 *vs*. 5.2%; at 12 months: 27.4 *vs*. 6.7%) (**Table 2**).

### Recurrence-Free Survival

**Figure 2:**
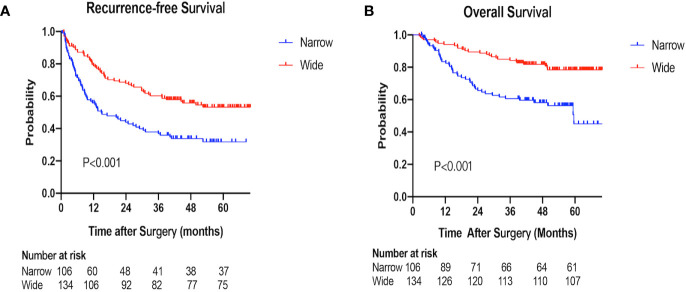
Recurrence-free survival (RFS) and overall survival (OS) of the narrow-margin and wide-margin groups. **(A)** Recurrence-free survival for patients with narrow and wide margin resection. **(B)** Overall survival for patients with narrow and wide margin resection.

**Figure 3:**
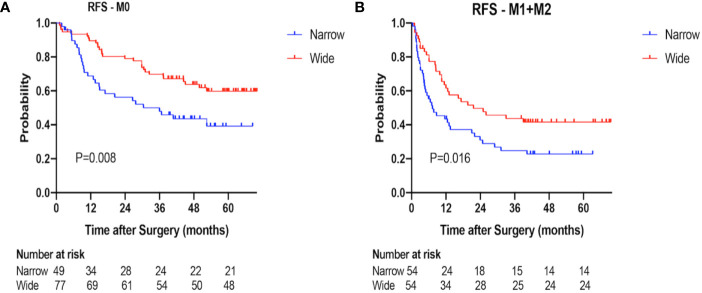
Recurrence-free survival (RFS) of the narrow-margin and wide-margin groups stratified based on microvascular invasion (MVI) status. **(A)** Recurrence-free survival in the subgroup of M0. **(B)** Recurrence-free survival in the subgroup of M1+M2.

**Figure 4:**
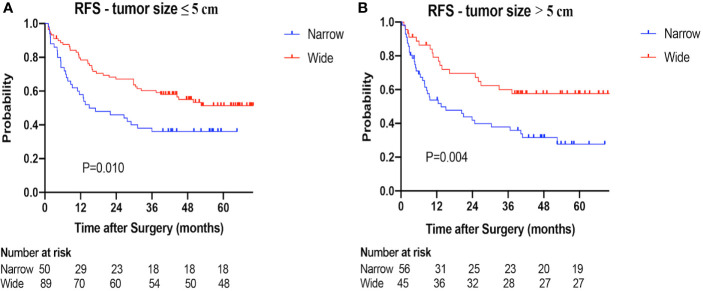
Recurrence-free survival (RFS) of the narrow-margin and wide-margin groups stratified based on tumor size. **(A)** Recurrence-free survival in the subgroup of tumor size ≤ 5 cm. **(B)** Recurrence-free survival in the subgroup of tumor size > 5 cm.

The 1-, 2-, 3-year RFS rates were 55.8, 43.9, 36.9% in the narrow-margin group and 78.7, 67.9, 60.2% in the wide-margin group, respectively (P<0.001; **Figure 2A**). In the multivariable analysis, narrow margin was significantly associated with worse RFS [wide *vs*. narrow, hazard ratio (HR) =0.608; 95% CI, 0.414–0.893, P=0.011]. Other independent predictors include HBs Ag, tumor capsule, MVI status, and extent of liver resection (**Table 3**). In the subgroup analysis based on MVI status (M0 *vs*. M1+M2), patients with narrow margin resection correlated with worse RFS, regardless of MVI status **(** **Figure 3** **)**. Similar results were also found in the subgroup analysis based on tumor size (≤ 5 cm *vs*. > 5 cm) **(** **Figure 4** **)**. The 1-, 2-, 3-year RFS rates for patients receiving TACE were 69.7, 51.8, 44.7%, respectively, comparing to 68.9, 59.1, 51.7% accordingly in the no adjuvant TACE group (**Supplementary Figure 1**).

**Table 3:** Prognostic factors of recurrence-free-survival (RFS) for patients with narrow and wide margin resection.

Variable	Univariate analysis		Multivariate analysis	
	HR (95% CI)	*P-*Value	HR (95% CI)	*P*-Value
Surgical margin (narrow/wide)	0.504 (0.356–0.715)	**<0.001**	0.608 (0.414–0.893)	**0.011**
Age (≤60/>60 years old)	0.850 (0.593–1.217)	0.374		
Gender (male/female)	0.724 (0.416–1.261)	0.254		
HBs Ag (negative/positive)	1.917 (1.200–3.062)	**0.006**	2.159 (1.302–3.580)	**0.003**
Cirrhosis (no/yes)	1.070 (0.726–1.579)	0.732		
Alcohol consumption (no/yes)	1.012 (0.714–1.435)	0.946		
AFP (≤20/>20ng/ml)	1.640 (1.144–2.351)	**0.007**	1.413 (0.968–2.063)	0.073
ALT (≤40/>40U/L)	1.904 (1.341–2.702)	**<0.001**	1.422 (0.973–2.076)	0.069
Child-Pugh class		0.166		
A5	Reference			
A6	0.876 (0.444–1.725)	0.701		
A7	2.970 (0.930–9.481)	0.066		
Tumor size (≤5/>5cm)	1.185 (0.835–1.683)	0.341	0.845 (0.570–1.253)	0.402
No. of tumor (single/multiple)	2.330 (1.535–3.536)	**<0.001**	1.281 (0.757–2.167)	0.356
Edmondson grades (I–II/III–IV)	1.287 (0.872–1.900)	0.204		
Tumor capsule (absent/present)	0.394 (0.267–0.581)	**<0.001**	0.447 (0.290–0.691)	**<0.001**
MVI classification		**<0.001**		**0.003**
M0	Reference		Reference	
M1	1.800 (1.214–2.671)	**0.003**	1.138 (0.693–1.868)	0.611
M2	3.364 (2.102–5.381)	**<0.001**	2.394 (1.382–4.147)	**0.002**
Unclear	0.336 (0.047–2.429)	0.280	0.233 (0.031–1.763)	0.158
Extent of liver resection (minor/major)	2.005 (1.415–2.841)	**<0.001**	1.586 (1.070–2.351)	**0.022**
pTNM stage		**<0.001**		0.560
I	Reference		Reference	
II	2.451 (1.611–3.729)	**<0.001**	1.487 (0.862–2.565)	0.154
III	4.978 (2.378–10.420)	**<0.001**	1.475 (0.510–4.264)	0.473
IVA	2.52 3(1.485–4.288)	**0.001**	1.326 (0.691–2.547)	0.396
Postoperative TACE (no/yes)	1.139 (0.769–1.686)	0.517		

Surgical resection, tumor number, tumor size, and variables with p value <0.01 in the univariable Cox analyses were retained for the multivariable Cox analysis. The foreparts of the parentheses were set as the reference groups in the univariable and multivariable Cox analysis.

HBs Ag, hepatitis B surface antigen; AFP, alpha-fetoprotein; ALT, alanine aminotransferase; MVI, microvascular invasion; pTNM stage, pathologic TNM stage; TACE, transcatheter arterial chemoembolization. P < 0.05 was indicated by bold values.

### Overall Survival

**Figure 5:**
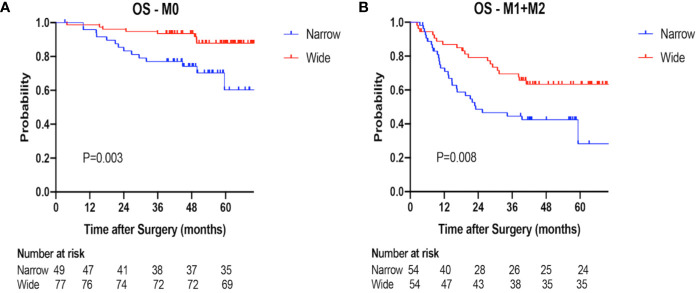
Overall survival (OS) of the narrow-margin and wide-margin groups stratified based on MVI status. **(A)** Overall survival in the subgroup of M0. **(B)** Overall survival in the subgroup of M1+M2.

**Figure 6:**
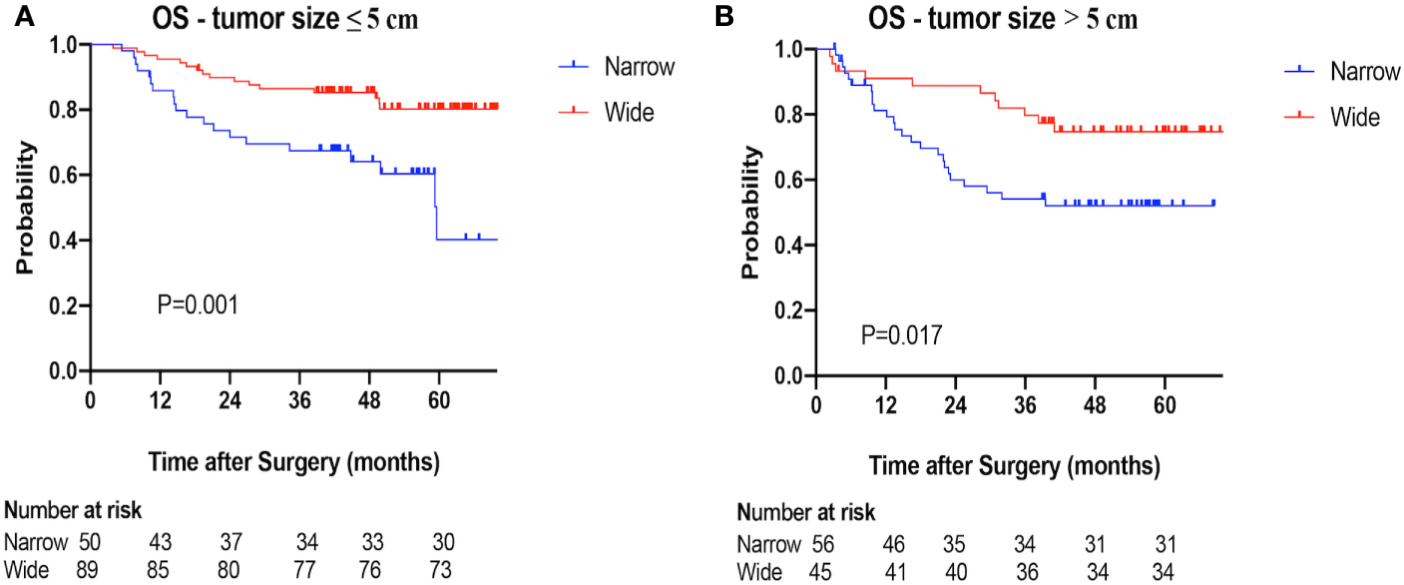
Overall survival (OS) of the narrow-margin and wide-margin groups stratified based on tumor size. **(A)** Overall survival in the subgroup of tumor size ≤ 5cm. **(B)** Overall survival in the subgroup of tumor size >5cm.

The 1-, 2-, 3-year OS rates were 83.5, 65.6, 60.6% in the narrow-margin group, and 94.0, 89.5, 84.2% in the wide-margin group, respectively (P<0.001; **Figure 2B**). In the multivariable analysis, narrow margin was significantly associated with worse OS (wide *vs*. narrow, HR=0.518; 95% CI, 0.308–0.871, P=0.013). Other independent predictors include Child-Pugh class, ALT level, tumor capsule, and MVI status (**Table 4**). In the subgroup analysis based on MVI status (M0 *vs*. M1+M2) and tumor size (≤ 5 cm *vs*. > 5 cm), patients with narrow margin resection correlated with worse OS, regardless of MVI status **(** **Figure 5** **)** or tumor size **(** **Figure 6** **)**. The 1-, 2-, 3-year OS rates for patients receiving TACE were 92.9, 89.3, 76.8%, respectively, comparing to 88.4, 75.9, 73.1% accordingly in the no adjuvant TACE group (**Supplementary Figure 1**).

**Table 4:** Prognostic factors of overall survival (OS) for patients with narrow and wide margin resection.

Variable	Univariate analysis		Multivariate analysis	
	HR (95% CI)	*P-*Value	HR (95% CI)	*P*-Value
Surgical margin (narrow/wide)	0.366 (0.226–0.591)	**<0.001**	0.518 (0.308–0.871)	**0.013**
Age (≤60/>60 years old)	0.960 (0.598–1.542)	0.866		
Gender (male/female)	0.543 (0.235–1.252)	0.152		
HBs Ag (negative/positive)	1.340 (0.747–2.402)	0.326		
Cirrhosis (no/yes)	1.091 (0.646–1.843)	0.744		
Alcohol consumption (no/yes)	0.923 (0.580–1.467)	0.734		
AFP (≤20/>20ng/ml)	2.042 (1.237–3.374)	**0.005**	1.611 (0.949–2.735)	0.077
ALT (≤40/>40U/L)	2.374 (1.494–3.771)	**<0.001**	1.941 (1.190–3.168)	**0.008**
Child-Pugh class		**<0.001**		**0.017**
A5	Reference		Reference	
A6	1.966 (0.975–3.966)	0.059	1.898 (0.882–4.082)	0.101
A7	8.140 (2.911–22.760)	**<0.001**	4.150 (1.355–12.708)	0.013
Tumor size (≤5/>5cm)	1.603 (1.010–2.545)	**0.045**	0.897 (0.531–1.514)	0.683
No. of tumor (single/multiple)	1.985 (1.138–3.462)	**0.016**	0.856 (0.414–1.771)	0.675
Edmondson grades (I–II/III–IV)	1.616 (0.984–2.654)	0.058	1.335 (0.765–2.332)	0.309
Tumor capsule (absent/present)	0.325 (0.201–0.527)	**<0.001**	0.421 (0.244–0.725)	**0.002**
MVI classification		**<0.001**		**<0.001**
M0	Reference		Reference	
M1	2.437 (1.373–4.325)	**0.002**	1.269 (0.633–2.544)	0.501
M2	7.194 (4.003–12.929)	**<0.001**	5.031 (2.510–10.085)	**<0.001**
Unclear	1.048 (0.141–7.784)	0.963	0.988 (0.123–7.929)	0.991
Extent of liver resection (minor/major)	2.440 (1.522–3.912)	**<0.001**	1.437 (0.839–2.462)	0.187
pTNM stage		**<0.001**		0.193
I	Reference		Reference	
II	3.743 (1.955–7.167)	**<0.001**	1.945 (0.874–4.326)	0.103
III	12.758 (5.000–32.555)	**<0.001**	4.547 (1.117–18.499)	**0.034**
IVA	4.454 (2.081–9.529)	**<0.001**	1.785 (0.722–4.416)	0.210
Postoperative TACE (no/yes)	0.921 (0.535–1.588)	0.768		

Surgical resection, tumor number, tumor size, and variables with p value <0.01 in the univariable Cox analyses were retained for the multivariable Cox analysis. The foreparts of the parentheses were set as the reference groups in the univariable and multivariable Cox analysis.

HBs Ag, hepatitis B surface antigen; AFP, alpha-fetoprotein; ALT, alanine aminotransferase; MVI, microvascular invasion; pTNM stage, pathologic TNM stage; TACE, transcatheter arterial chemoembolization. P < 0.05 was indicated by bold values.

### Patterns of Recurrence for Positive Margin Cases With Postoperative Stereotactic Body Radiotherapy

Finally, nine patients who underwent postoperative SBRT after positive margin resection were included in this analysis. The postoperative radiotherapy was given to a total dose of 35Gy/5F to 50Gy/5F (median, 40Gy/5F). The median interval between operation and initiation of postoperative SBRT was 51 days. The incidence and pattern of recurrence have been detailed in **Table 2**. Median follow-up period is 15.1 (range 6.8–38.6) months. During the follow-up periods, two patients (2/9, 22.2%) had documented tumor recurrence, with one patient (1/9, 11.1%) developed intrahepatic multiple-nodule recurrence at 7.9 months after surgery, and the other case had intrahepatic single-nodule recurrence far away from the resection margin at 7.5 months post-operation.

Patients with positive margin resection plus SBRT or wide-margin resection showed a significantly lower incidence of total recurrence than that with narrow-margin resection (positive plus SBRT *vs*. wide *vs*. narrow: 22.2 *vs*. 44.0 *vs*. 65.1%). Regarding the pattern of marginal recurrence, a large numerical difference was found among the three groups. Patients with positive margin resection plus SBRT or wide-margin resection experienced a significantly lower rates of marginal recurrence than that with narrow-margin resection (positive plus SBRT *vs*. wide *vs*. narrow: 0.0 *vs*. 4.5 *vs*. 20.8%). With regard to the pattern of intrahepatic multiple-nodule recurrence, patients with positive margin resection plus SBRT or wide-margin resection showed a significantly lower rate of recurrence than that with narrow margin resection (positive plus SBRT *vs*. wide *vs*. narrow: 11.1 *vs*. 11.9 *vs*. 32.1%).

The time interval to recurrence significantly differed among the three groups. Compared with patients with positive margin plus SBRT and with wide margin resection, those with narrow margin resection had a higher rate of recurrence within 12 months after surgery (positive plus SBRT *vs*. wide *vs*. narrow: 22.2 *vs*. 20.9 *vs*. 43.4%).

The toxicity associated with postoperative SBRT is summarized in **Table 5**. Grade 1 myeloid suppression was the most common toxicity encountered during SBRT, followed by grade 1 liver enzyme (44.4%) and bilirubin (33.3%) elevation. One (11.1%) patient combined cirrhosis history experienced grade 2 thrombocytopenia. No grade 3 and 4 toxicities were seen. No radiation induced liver disease was encountered.

**Table 5:** Toxicity from stereotactic body radiotherapy (SBRT) in patients with positive margin.

Toxicity grade (CTCAE)	0	1	2	3	4
Myeloid suppression					
Leukopenia	4 (44.4%)	5 (55.6%)	0 (0%)	0 (0%)	0 (0%)
Lymphopenia	8 (88.9%)	1 (11.1%)	0 (0%)	0 (0%)	0 ()%)
Thrombocytopenia	7 (77.8%)	1 (11.1%)	1 (11.1%)	0 (0%)	0 (0%)
Constitutional symptoms					
Fatigue	7 (77.8%)	2 (22.2%)	0 (0%)	0 (0%)	0 (0%)
Fever (tympanic)	9 (100.0%)	0 (0%)	0 (0%)	0 (0%)	0 (0%)
Insomnia	9 (100.0%)	0 (0%)	0 (0%)	0 (0%)	0 (0%)
Abdominal pain	9 (100.0%)	0 (0%)	0 (0%)	0 (0%)	0 (0%)
Gastrointestinal					
Anorexia	9 (100.0%)	0 (0%)	0 (0%)	0 (0%)	0 (0%)
Diarrhea	9 (100.0%)	0 (0%)	0 (0%)	0 (0%)	0 (0%)
Constipation	9 (100.0%)	0 (0%)	0 (0%)	0 (0%)	0 (0%)
Nausea	8 (88.9%)	1 (11.1%)	0 (0%)	0 (0%)	0 (0%)
Vomiting	8 (88.9%)	1 (11.1%)	0 (0%)	0 (0%)	0 (0%)
Metabolic/laboratory					
Albumin	9 (100.0%)	0 (0%)	0 (0%)	0 (0%)	0 (0%)
Liver transaminase (ALT or AST)	5 (55.6%)	4 (44.4%)	0 (0%)	0 (0%)	0 (0%)
Bilirubin	6 (66.7%)	3 (33.3%)	0 (0%)	0 (0%)	0 (0%)
Radiation induced liver disease (RILD)	9 (100.0%)	0 (0%)	0 (0%)	0 (0%)	0 (0%)

## Discussion

This study analyzed the association of surgical margin with recurrence pattern. Higher rate of recurrence (especially the pattern of marginal recurrence) and lower overall survival were found in HCC with narrow margin resection compared to that with wide margin resection. The addition of SBRT to patients with positive surgical margin reduced the recurrences, particularly the pattern of marginal recurrence. Our findings implicate the potential feasibility of postoperative SBRT for patients with narrow margin resection.

Narrow margin resection may be the most appropriate procedure for HCC adjacent to major vessels because the premise for survival is the conservation of more normal liver parenchyma (17). Unfortunately, narrow margin resection has been reported to contribute to poor survival outcomes due to the high frequency of recurrence and the clinical significance remains controversial (9, 11, 26–29). Shi et al. investigated the influence of the width of resection margin on postoperative recurrence and found that narrow margin group had significantly higher rate of recurrence than wide margin group (52.4 *vs*. 36.5%, P=0.037), and wide margin resection efficaciously decrease recurrence and improve survival (11). Chau et al. also showed a similar results (narrow and wide: 61.3 *vs*. 36.5%) (29). However, Poon et al. reported that the width of margin did not influence the postoperative recurrence rates (narrow and wide: 64.0 *vs*. 59.4%, P=0.943) (9). It is worth noting that these controversial results might be due to the heterogeneity of tumor characteristics and surgery procedures, such as cirrhosis (Shi’s and Poon’s: 80.5 *vs*. 46.2%), resection extent ≥3 segments (Shi’s and Poon’s: 10.1 *vs*. 65.3%), and preoperative transfusion (Shi’s and Poon’s: 26.6 *vs*. 58.0%). Our results were consistent with the former, showing that HCC patients with narrow margin resection had higher rate of recurrence compared to those with wide margin resection (P=0.001), and multivariable analysis showed that narrow margin was significantly associated with worse RFS and OS, indicating that narrow margin resection alone is insufficient for tumor eradication and adjuvant therapy is imperative to reduce the risk of recurrence. Identifying the failure patterns help to further guide appropriate management of postoperative therapy.

In our study, the intrahepatic recurrence patterns were defined as marginal, intrahepatic single-nodule and multiple-nodule recurrences. We found that patients with narrow margin resection experienced a higher marginal recurrence, as well as intrahepatic multiple nodules recurrence. The findings were consistent with previous reports (11, 29). For example, in the study conducted by Shi et al. all marginal recurrences were observed in narrow margin group and multinodular recurrence was also significantly higher than that in wide margin group (P=0.018) (11). Taken together, postoperative marginal recurrence for patients with narrow margin seems to be common, about 21% of the narrow-margin HCC patients recur within 1cm from the surgical margin in our study, suggesting that adjuvant local therapy might improve the local-regional control.

Higher intrahepatic multiple-nodule recurrences were also seen in patients with narrow margin, and associated with higher marginal recurrence. For multiple-nodule recurrences, it is difficult to identify which lesion occurred first. According to the previous study, micro-metastasis was commonly remnant from resection margin within 1 cm, surgical margin recurrence might occur first, then spread to the whole residual liver *via* portal vein branches (4, 6, 30, 31). Therefore, we cautiously speculate that marginal recurrence may be one of driver factors of intrahepatic multiple recurrence, and multifocal recurrence might be prevented or mitigated if liver marginal recurrence could be well controlled by adjuvant local therapy. However, margin status alone is an insufficient explanation as to why intrahepatic other regions yields recurrence located over 1 cm from the area of resection, inherent imbalance in the tumor characteristics, such as microvascular invasion status, were also related to the recurrence pattern (32).

Our multivariate analysis showed that MVI were negatively related to RFS and OS. Previous studies reached similar conclusion that MVI was closely related to early recurrence and dismal prognosis (27, 30, 33, 34). Additionally, absence of tumor capsule was significantly correlated to poorer long-term outcomes, which was in line with the previous studies (8, 35–38). Therefore, adjuvant therapy may be useful for those with high risk factors.

We also evaluated the influence of postoperative SBRT on the incidence and pattern of recurrence. The rate of marginal and multiple-nodule recurrences in the group of positive-margin plus SBRT was comparable to that of wide-margin group, and better than that of narrow-margin group, suggesting that the addition of postoperative local radiotherapy could provide an improved local control and mitigate the recurrence risk due to insufficient surgical margin. In spite of the small number of cases, our data does highlight the efficacy of postoperative SBRT in reducing the recurrence risk for patients with positive margin, and presumably a potential effect on narrow margin resection.

Considering the pattern of marginal recurrence itself occurs frequently and it may, subsequently, induce the occurrence of intrahepatic multiple-nodule in patients with narrow margin resection, postoperative local radiotherapy may provide a favorable outcome through its role of local-regional control. To date, with the improvement of radiotherapy techniques, such as intensity modulated radiotherapy (IMRT) and SBRT, a significantly reduced radiation-induced toxicity and increased radiotolerance of normal tissue were obtained (39, 40). A few prospective studies have been carried out to demonstrate the feasibility and advantages of adjuvant radiotherapy (32, 41, 42). For instance, Wang et al. reported that the efficacy of receiving IMRT following narrow margin resection was comparable to that of wide margin hepatectomy and superior to those of narrow margin resection alone, and none of the patients receiving IMRT developed radiation-induced liver disease (42). Yu et al. revealed that patients who underwent three-dimensional conformal radiotherapy following narrow margin resection yielded recurrence-free survival outcomes significantly superior to those of narrow margin resection alone, in patients with HCCs smaller than 5cm (41). Additionally, one clinical trial showed that for HCC patients with MVI, adjuvant three-dimensional conformal or IMRT could result in better survival outcomes than TACE or conservative therapy following narrow margin hepatectomy, considering radiotherapy could eliminate residual micro-metastasis-foci in the remnant liver (32). However, there is a lack of exploration for the efficacy of adjuvant SBRT. SBRT has shown encouraging rates of local control for HCC (43). Compared with standard fractionation radiation, SBRT can achieve more precise delivery of high-dose radiation beams to the lesion, obtaining a much smaller target volume. Meanwhile, it could be finished in a short period which can bring more convenience to patients (25, 40). In our study, the total recurrence rate in the patients with positive-margin plus SBRT (2/9, 22.2%) was satisfied, comparing by patients with negative-margin resection alone (128/240, 53.3%). The marginal recurrence rate with SBRT was relatively lower. Given these compelling results, postoperative radiotherapy may represent an innovative strategy to optimize the amelioration of tumor recurrence. A further large-sample clinical data is warranted to demonstrate the benefits of adjuvant radiotherapy in patients with narrow margin resection, considering the small sample size of above-mentioned studies.

In summary, we found that HCC patients following narrow margin hepatectomy had a higher recurrence rate and poorer prognosis, with 20.8% patients developed marginal recurrence. Postoperative SBRT treatment for patients with positive margin showed low recurrence rate and no marginal recurrence was found. There is a limitation of this study with small patient samples received postoperative SBRT and short patient follow-up. Therefore, high-quality multi-center prospective studies are needed to further confirm the efficacy of adjuvant radiotherapy.

## Conclusion

Patients with narrow margin were associated with higher recurrence (especially for the pattern of marginal recurrence) and worse survival outcomes than those with wide resection margin. Postoperative local treatment, such as radiotherapy, might bring potential benefit for these patients.

## Data Availability Statement

The raw data supporting the conclusions of this article will be made available by the authors, without undue reservation.

## Ethics Statement

The studies involving human participants were reviewed and approved by the Institutional Review Board of Zhejiang University School of Medicine. The patients/participants provided their written informed consent to participate in this study.

## Author Contributions

LL and YS wrote the article and analyzed the data and statistics. QY and YG analyzed the data and statistics. LZ and XZ reviewed and edited the article. RY and JL were responsible for image evaluation and clinical data analysis. QW and SW designed, revised, and supervised the writing and concept of the article. All authors read and approved the final manuscript. All authors contributed to the article and approved the submitted version.

## Funding

This work was supported by the National Natural Science Foundation of China (No. 81572952).

## Conflict of Interest

The authors declare that the research was conducted in the absence of any commercial or financial relationships that could be construed as a potential conflict of interest.

## Supplementary Material

Supplementary Figure 1 Recurrence-free survival (RFS) and Overall survival (OS) of patients with or without postoperative TACE. **(A)** Recurrence-free survival for patients with TACE and without TACE. **(B)** Overall survival for patients with TACE and without TACE.

## Abbreviations

CI, confidence interval; GTV, gross target volume; HCC, hepatocellular carcinoma; HR, hazard ratio; IMRT, intensity modulated radiotherapy; ITV, internal target volume; MVI, microvascular invasion; OAR, organs at risk; OS, overall survival; PTV, planning target volume; RFS, recurrence-free survival; RILD, radiation induced liver disease; SBRT, stereotactic body radiotherapy; TACE, transcatheter arterial chemoembolization.
