# Supplementary material for: Narrow-Margin Hepatectomy Resulted in Higher Recurrence and Lower Overall Survival for R0 Resection Hepatocellular Carcinoma
Source: Front Oncol. 2021 Jan 21;10:610636. doi: 10.3389/fonc.2020.610636 (PMC7859433; doi:10.3389/fonc.2020.610636)
Supplement: Supplementary Figure 1 — Recurrence-free survival (RFS) and Overall survival (OS) of patients with or without postoperative TACE. (A) Recurrence-free survival for patients with TACE and without TACE. (B) Overall survival for patients with TACE and without TACE. [file Image_1.pdf]

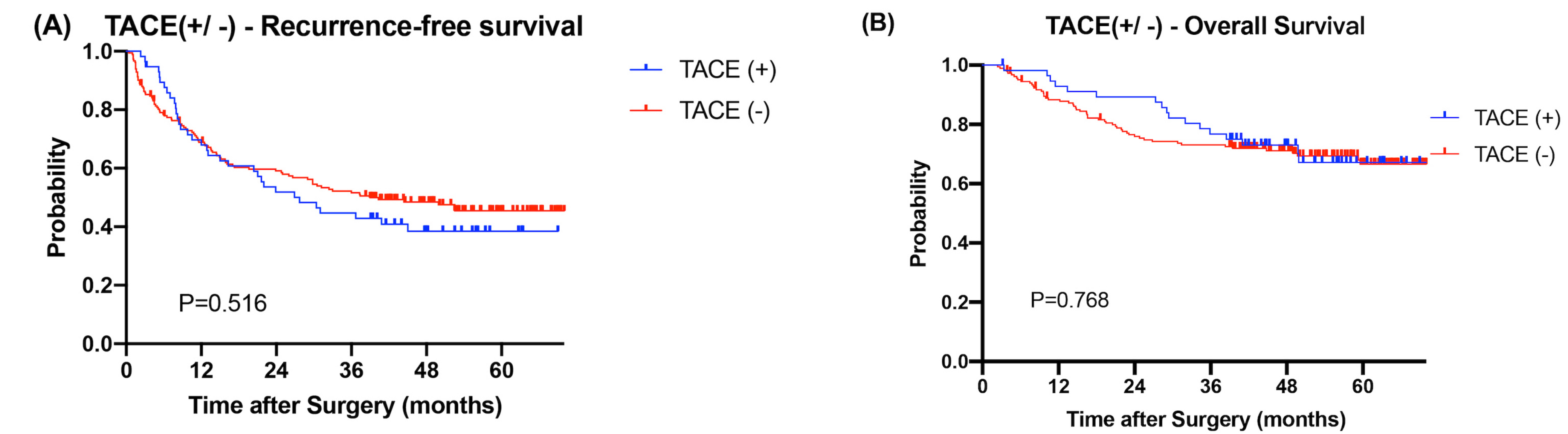

Number at risk (A)

TACE (+)	57	39	30	26	23	23
TACE (-)	183	127	110	97	92	89

Number at risk (B)

TACE (+)	57	53	51	44	42	40
TACE (-)	183	162	140	135	132	128
