# Supplementary material for: Narrow-Margin Hepatectomy Resulted in Higher Recurrence and Lower Overall Survival for R0 Resection Hepatocellular Carcinoma
Source: Front Oncol. 2021 Jan 21;10:610636. doi: 10.3389/fonc.2020.610636 (PMC7859433; doi:10.3389/fonc.2020.610636)
Supplement: Supplementary file 2 [file Table_1.docx]

**Suppl. Table 1. Demographic and clinicopathological characteristics** **of patients** **with or without postoperative TACE**

| Variable | TACE (+) | TACE (-) |  |
| --- | --- | --- | --- |
|  | （n=57） | (n=183) | *P*-Value |
| Age  ≤60 y old  >60 y old | 36 (63.2%)  21 (36.8%) | 112 (61.2%)  71 (38.8%) | 0.876 |
| Gender  Male  Female | 46 (80.7%)  11 (19.3%) | 162 (88.5%)  21 (11.5%) | 0.178 |
| HBs Ag  Positive  Negative | 44 (77.2%)  13 (22.8%) | 139 (76.0%)  44 (24.0%) | 1.000 |
| Cirrhosis  Yes  No | 40 (70.2%)  17 (29.8%) | 134 (73.2%)  49 (26.8%) | 0.734 |
| Alcohol consumption Yes  No | 29 (50.9%)  28 (49.1%) | 103 (56.3%)  80 (43.7%) | 0.543 |
| AFP  ≤20 ng/ml  >20 ng/ml | 25 (43.9%)  32 (56.1%) | 78 (42.6%)  105 (57.4%) | 0.879 |
| ALT  ≤40 U/L  >40 U/L | 39 (68.4%)  18 (31.6%) | 115 (62.8%)  68 (37.2%) | 0.528 |
| TBIL  ≤17.1umol/L  >17.1umol/L | 35 (61.4%)  22 (38.6%) | 124 (67.8%)  59 (32.2%) | 0.423 |
| ALB  ≤35 g/L  >35 g/L | 9 (15.8%)  48 (84.2%) | 13 (7.1%)  170 (92.9%) | 0.064 |
| PT% <75 75-100 >100 |  |  | 0.188 |
|  | 5 (8.8%) | 10 (5.5%) |  |
|  | 42 (73.7%) | 120 (65.6%) |  |
|  | 10 (17.5%) | 53 (29.0%) |  |
| Child-Pugh class  A5 A6 B7 |  |  | 0.327 |
|  | 52 (91.2%) | 164 (89.6%) |  |
|  | 3 (5.3%) | 17 (9.3%) |  |
|  | 2 (3.5%) | 2 (1.1%) |  |
| Tumor size  ≤5 cm  >5 cm | 30 (52.6%)  27 (47.4%) | 109 (59.6%)  74 (40.4%) | 0.361 |
| No. of tumor  Single  Multiple | 47 (82.5%)  10 (17.5%) | 158 (86.3%)  25 (13.7%) | 0.520 |
| Edmondson grades  I-II  III-IV | 39 (68.4%)  18 (31.6%) | 143 (78.1%)  40 (21.9%) | 0.157 |
| Tumor capsule  Present  Absent | 46 (80.7%)  11 (19.3%) | 148 (80.9%)  35 (19.1%) | 1.000 |
| MVI classification |  |  | 0.144 |
| M0 | 25 (43.9%) | 101 (55.2%) |  |
| M1 | 24 (42.1%) | 49 (26.8%) |  |
| M2 | 6 (10.5%) | 29 (15.8%) |  |
| Unclear | 2 (3.5%) | 4 (2.2%) |  |
| Extent of resection  Minor  Major | 29 (50.9%)  28(49.1%) | 106 (57.9%)  77 (42.1%) | 0.363 |
| pTNM stage I II^*^ III IVA | 17 (29.8%) 25 (43.9%) 5 (8.8%) 10 (17.5%) | 77 (42.1%)  75 (41.0%)  5 (2.7%)  26 (14.2%) | 0.118 |
| Surgical margin |  |  | 0.446 |
| Narrow | 28 (49.1%) | 78 (42.6%) |  |
| Wide | 29 (50.9%) | 105 (57.4%) |  |
| Median RFS (m) | 27.7 | 39.0 | 0.516 |
| Median OS (m) | Undefined | Undefined | 0.768 |
| Values in parentheses are percentages and P<0.05 was indicated by bold values.  Abbreviations：HBs Ag = hepatitis B surface antigen; AFP = alpha-fetoprotein; ALT = alanine aminotransferase; TBIL = total bilirubin; ALB = albumin; PT = prothrombin time; MVI = microvascular invasion; pTNM stage = pathologic TNM stage; TACE = transcatheter arterial chemoembolization;  ^*^5 cases that T stage (T1b or T2) was undefined because of unclear MVI status were belonged in category of T2 stage. | | | |
